# Consumption Analysis of Smartphone based Fall Detection Systems with Multiple External Wireless Sensors

**DOI:** 10.3390/s20030622

**Published:** 2020-01-22

**Authors:** Francisco Javier González-Cañete, Eduardo Casilari

**Affiliations:** Departamento de Tecnología Electrónica, Universidad de Málaga, ETSI Telecomunicación, 29071 Málaga, Spain; ecasilari@uma.es

**Keywords:** fall detection system, inertial sensors, smartphones, accelerometers, gyroscopes, Android, battery consumption

## Abstract

Fall Detection Systems (FDSs) based on wearable technologies have gained much research attention in recent years. Due to the networking and computing capabilities of smartphones, these widespread personal devices have been proposed to deploy cost-effective wearable systems intended for automatic fall detection. In spite of the fact that smartphones are natively provided with inertial sensors (accelerometers and gyroscopes), the effectiveness of a smartphone-based FDS can be improved if it also exploits the measurements collected by small low-power wireless sensors, which can be firmly attached to the user’s body without causing discomfort. For these architectures with multiple sensing points, the smartphone transported by the user can act as the core of the FDS architecture by processing and analyzing the data measured by the external sensors and transmitting the corresponding alarm whenever a fall is detected. In this context, the wireless communications with the sensors and with the remote monitoring point may impact on the general performance of the smartphone and, in particular, on the battery lifetime. In contrast with most works in the literature (which disregard the real feasibility of implementing an FDS on a smartphone), this paper explores the actual potential of current commercial smartphones to put into operation an FDS that incorporates several external sensors. This study analyzes diverse operational aspects that may influence the consumption (as the use of a GPS sensor, the coexistence with other apps, the retransmission of the measurements to an external server, etc.) and identifies practical scenarios in which the deployment of a smartphone-based FDS is viable.

## 1. Introduction

Aging is one of the greatest challenges for public health systems, at least in industrialized countries. For example, according to the last ageing report of the EU, the proportion of the European population in the working age (15–64) will decrease from 65.16% in 2016 to 56.15% in 2070, while the life expectancy at birth is expected to augment from 78.3 in 2016 to 86.1 in 2070 (for males) and from 83.7 in 2016 to 90.3 in 2070 (for females) [1]. In this respect, falls represent one of the greatest threats to the wellbeing and autonomy of older people. The data reported by the WHO (World Health Organization) reveal that approximately one third of persons aged over 65 experience a fall every year, while this percentages reaches 50% for people over 85 [2]. In 2014 alone, older persons in the USA suffered 29 million falls, resulting in seven million injuries and an estimated Medicare cost of $31 billion [3]. A total of 40% of admissions of older Medicare population in USA hospitals were associated to fall-related injuries, which provoked an average hospital stay of 11.6 days [4].

After a collapse, a high number of fallers (47%) (even those uninjured) found themselves unable to get up unassisted [5] because of their lack of physical fitness [6]. Unfortunately, a long lie on the floor after a fall is straightforwardly connected to co-morbidities such as pneumonia, dehydration, hypothermia and sores that increase by 50% the probability of death within the following six months [7,8]. Thus, the quick delivery of assistance after a fall is a key element to reduce hospitalization and physical and psychological disorders (such as FoF—Fear of Fall) that directly affect the self-confidence and capability of the seniors to live independently. As a result of this situation, the development of effective automatic Fall Detection Systems (FDS) has become a relevant research topic in the field of telemedicine and telemonitoring applications in the last decade.

The generic goal of an FDS is to monitor a certain patient (or user) in a permanent way so that whenever a fall is suspected, an alarm (phone call, acoustic alert, text message, etc.) is seamlessly generated without the intervention of the patient (since they can remain unconscious on the ground). The alarm is then automatically transmitted to a remote telemonitoring point or authorized personnel (clinic, relatives, call center, etc.), who can locate the faller and rapidly provide medical assistance.

An FDS is programmed to discriminate possible falls from the rest of the user movements, commonly referred to as Activities of Daily Living (ADL). Thus, the performance of an FDS (which can be pictured as a binary classification system) is evaluated by assessing its effectiveness to recognize falls when they occur (sensitivity) and the ability (specificity) to avoid false alarms (i.e., to misinterpret an ADLs as a fall.)

In a generic way, FDSs can be categorized into two major typologies. On the one hand, context-oriented systems (CAS) propose to identify falls based on the signals of a series of environmental sensors (pressure and vibration sensors under carpets—or ‘smart floors’—infrared motion sensors, microphones, depth cameras, radars, ultrasonic transducers, etc.) located in a specific area in the vicinity of the user (residences, nursing homes, etc.). Apart from their higher costs of installation, equipment adjustment and maintenance, the main disadvantage of these contextual systems is that their operation is limited to a very particular supervised area. Therefore, the tracking of the users ceases as they abandon this specific monitoring zone. Moreover, the detection based on environmental sensors is prone to errors caused by external elements, such as noises, room lightning changes, interaction of other individuals, falling objects or pets, shadowing caused by the alteration in the position of furniture, etc. In addition, the use of audiovisual sensors may compromise the privacy of the patients.

On the other hand, wearable FDSs overcome these shortcomings since the employed sensors (mostly inertial measurements units) are directly attached to the body (or clothes) of the patient that must be tracked. Thus, they only monitor variables (such as acceleration or angular velocity of the body) that are unambiguously associated to the patient mobility. As wearable systems can be easily endowed with long-range wireless interfaces (such as mobile communications), they can operate almost ubiquitously (at least in urban settings), which gives the user much greater freedom of movement. Furthermore, this type of FDSs can benefit from the plummeting costs of MEMs technology as well as from the widespread popularity and commercial expansion of certain wearables. On the contrary, wearable systems also exhibit some disadvantages related to the ergonomics of the FDS and the user comfort and particularly, to the autonomy of the battery-powered elements that make up the fall detector.

A wearable FDS basically consists of three elements:

- A sensing sub-system comprising one or several inertial sensors (normally accelerometers, but also gyroscopes and magnetometers), which are usually complemented by a GPS tracker and in some particular cases, biosensors (e.g., an ECG sensor).
- A ‘decision maker’ section or ‘decision core’, which processes the signals captured by the previous block and generates a binary decision (fall/ADL) about the present movement by applying a certain detection algorithm.
- A communication module, which is in charge of emitting a local (and if necessary, a remote) alert message as soon as a movement is identified as a fall.

Smartphones have been judged as an attractive solution to implement inexpensive wearable FDS as they can easily carry out these three functionalities: they natively embed mobile and Wi-Fi communications as well as an Inertial Measurement Unit—IMU—(or, at least, an accelerometer) while they offer enough computing power to deploy detection algorithms capable of producing real-time decisions based on the measurements sampled by the built-in IMU. In this respect, considering the nature of the device where these three activities take place, wearable FDSs can be, in turn, classified into three great groups:

- Systems based on a Specific Device (SD): this category encompasses those architectures that do not consider the use of a smartphone and are completely put into effect on a single purpose-designed device. In this case, the wearable sensing mote must integrate both the system decision core and the communication module. Most commercial fall detection products (sold in the form of a pendant, a bracelet or a wrist garment) follow this scheme [9,10]. Despite the undoubted advances in the field of wearable devices during recent years, these ‘self-contained’ platforms purposely designed to track the movements may still pose a challenge in terms of the computation capabilities and battery consumption that are required to implement the detection algorithm in the mote. Apart from this, these ‘onboard’ detectors entail an extra monthly expense due to the mobile connections that are needed for ubiquitous monitoring (e.g., the cost of acquiring and maintain a SIM card to enable automatic alarm phone calls or text messages).
- Smartphone-only architectures, which integrate all the three functions of the FDS (sensing, detecting and alarming) into a unique application running in a smartphone. Under this scheme, the FDS can be put into action as a simple app supported by a widespread personal device. Consequently, it does not entail any additional costs or hardware. Smartphones, though, are not conceived to operate as corporal sensing units. Wearables IMUs, which can be easily adjusted and even integrated into the clothes as simple garments, are clearly preferable to smartphones in terms of ergonomics and portability (weight and size), ease of use and cost. When utilized as a sensor, the comfortability, non-intrusiveness and usability of a smartphone is notably compromised as long as it has to be firmly attached to the body in an unnatural location for a more accurate characterization of the mobility of the body mass center. For example, to guarantee a proper characterization of the user mobility, in some cases, the smartphone (or even the smartwatch) is strapped to the chest of the user [11], which is a quite an uncomfortable position to operate normally with these general purpose devices.
- ‘Hybrid’ systems. In this group we include FDSs that combine a smartphone and one or several external wearable mobility sensors. Under this approach, the activity of the external units (lightweight, small, general purpose motes intended to track the user movements) is exclusively focused on the sensing operation. A low-power low-range wireless protocol is employed to transmit the captured signals from the sensor to the phone, which is only in charge of implementing the detection algorithm and sending the alarm in case of falls. As the phone is released from acting as a sensor, it can be transported in an arbitrary position (pocket, bag, etc.) as long as it is guaranteed that is not far enough (up to several meters) to interrupt the low range communications with the external units. In this regard, Bluetooth Low Energy (BLE) is considered to be a good alternative to enable the communications between the smartphone and the sensors. The reasons for this election are twofold: firstly, it is the only low-power wireless technology that is factory-fitted integrated into most existing smartphone models. Secondly, BLE has been shown to outperform the capabilities of other low consumption standards (such as IEEE 802.15.4) to deploy high data rate Body Area Networks [12].

Hybrid systems may simultaneously benefit from the low cost, small size and portability of existing IMUs with low range transmission interfaces as well as from the wireless interfaces, mobile connectivity and computing capability of present smartphones. Nonetheless, the practical problems associated to deploy a hybrid architecture on a smartphone are rarely addressed by the related literature. To close this gap, this work systematically assesses the impact of implementing a fall detection application on different smartphones with diverse versions of the Android operating system. To this end, this study thoroughly analyses the energy drain caused by the permanent connection to the external units as well as by the use of GPS. Another operational aspect that is usually neglected by the recent work on smartphone-based FDS is the fact that the detection app should neither affect nor be affected by the conventional use of these personal devices. Thus, this paper also evaluates the coexistence of the detection app with other typical high power-consuming functionalities of the smartphones.

The general objective of this paper is twofold: on the one hand, following the previous taxonomy of the current topologies of wearable FDSs, a critical review of those works that have evaluated the practical aspects (battery, consumption of hardware resources) of the implementation of these topologies in real devices is offered. Secondly, a detailed and multidimensional empirical analysis of the technological viability of using smartphones as the central element of hybrid architectures is presented.

This work is organized as follows: after the introduction in Section 1, Section 2 presents the state-of-the-art. Section 3 describes the employed testbed while Section 4 and Section 5 present and discuss the results obtained from the systematic evaluation of different configurations of a hybrid FDS system. Finally, Section 6 summarizes the main conclusions of the work.

## 2. Related Literature

### 2.1. Consumption in Wearable FDSs Based on a Specific Device (not Employing Smartphones)

Energy consumption has been studied for some prototypes that do not consider a smartphone as the central node or gateway of the architecture. For example, Nguyen et al. investigated, in [13], the impact of diverse parameters (sampling rate, communication interface, transmission protocol and data rate) on the power consumption experienced by a wearable sensor node specifically designed for fall detection purposes. The results show that for the worst case, the prototype can operate during a period of 76 h of constant monitoring without recharging its 1000 mAh battery. Wireless communications are provided through an RF (Radio Frequency) module that connects to a line-powered gateway, so that the tracking of the user is restrained to a particular home environment (in contrast with the almost ubiquitous monitoring offered by smartphone-based architectures). Additionally, the threshold-based detection technique is very simplistic to allow its implementation in the low-power sensing mote (in fact, the performance of the algorithm is not systematically evaluated with experimental users).

Important practical limitations arise when commercial wearables and their associated platforms (such as cloud-computing platforms) are utilized for continuous monitoring under conventional ‘free-living’ conditions for gait assessment and fall detection [14]. In an FDS application, sensing and wireless communications are always the two leading factors for battery drain of the wearable motes. In this regard, the study by Williamson et al. in [15] reveals that the current required by microelectromechanical IMUs is proportional to the sampling frequency, the resolution and SNR of the sensor, while the consumption of the most employed low-power wireless standard (Bluetooth Low Energy) is also strongly determined by the dynamics of the transmissions (i.e., advertising or connection event interval).

As it refers to the power required by the decision algorithm, the work by Wang et al. in [16] analyzes the energy consumed by a decision tree fall detection algorithm, which is put into operation on a specific low power sensing mote installed on a pendant worn on a lanyard around the neck. By comparing a set of ten input features derived from different statistics extracted from the accelerometer and gyroscope signals, the study concludes that a remarkable power saving (of up to 75%) can be achieved if the features are carefully selected.

Rathi et al. described, in [17], one of the few designs of a wearable FDS that is thoroughly conceived to optimize energy consumption. The proposed prototype, which incorporates three sensors (on chest, waist and sacral region) with embedded accelerometers and gyroscopes, is aimed at discriminating the pre-fall phase from the movements of the user. The applied algorithm is founded on a trivial thresholding strategy technique, which produces a relatively low detection rate during the test with volunteers. Furthermore, the system does not incorporate any alarming module. Thus, whenever a fall is suspected in a sensor, it just records the event in its internal SD memory. In fact, the authors propose providing the motes with wireless interfaces as a future research.

Narayanan et al. presented, in [18], a specific sensing wearable node (which is transported as a pendant on a lanyard around the neck) intended to detect falls based on the joint information provided by an accelerometer and a barometer. The node is designed to minimize the consumption. In fact, after analyzing the behavior of the mote during a one-week free-living trial, the battery is estimated to be operative for more than 600 days. One of the key elements to reduce the battery drain is to diminish the sampling rate of the accelerometer to 6 Hz, which could explain the low performance metrics that are achieved (with a specificity lower than 90%). In addition, the module employs short-range communications by using a 2.4 GHz transceiver. Thus, monitoring is constrained to domestic scenarios.

Two similar prototypes which are optimized to reduce the consumption are presented in the work by Ren et al. [19] (exhibiting an estimated battery lifetime of up to 21.7 days) and by Yuan et al. in [20] (with a battery lifespan of 78.7 days). In both cases, the employed low-power technology (802.15.4) of the transceiver restricts the transmission range to some meters so that the operation of the system is limited to home-based environments in which a RF-receiver can be located in the vicinity of the user.

### 2.2. Consumption in Smartphone-Only Based Architectures

The typical battery capacity of smartphones has been continuously increasing during the last decade: from 950 mAh in 2008 to 1500 mAh in 2013 [21] and up to 3600 mAh for top-end terminals in 2019. However, in general, the growing emergence of new devices and apps with innovative possibilities has not been accompanied by a similar evolution of battery technology [22]. To a large extent, the battery has become the ’bottleneck’ of the usability of smartphones so that many devices are designed to guarantee only one day (or even only some hours) of usage under heavy use [23]. A battery lifetime lower than one day can be inadmissible for many medical monitoring applications, as long as the tracking process would require to be interrupted before bedtime to replace or recharge the batteries. If an extra fall detection app is running permanently on the phone, this typical lifespan of 24 h could be lessened. In fact, according to certain questionnaires fulfilled by the volunteers of a real life trial of an FDS [24], FDSs should be operative for at least six months with no battery recharge in order to be considered satisfactory by the users (an autonomy which is obviously far from the actual features of current devices).

In spite of its importance, the problem of battery drain in standalone smartphone-based FDSs has been addressed by a relatively low number of studies in the related literature. In some of these studies, the study of battery consumption is just contemplated as a future work in an ongoing research [25,26].

Luque et al. claimed, in [27], that a major problem may appear in the smartphone-only architecture that they propose if a mobile app is permanently employed for monitoring the mobility of the user. The difficulties derived from battery depletion and lack of computing capabilities to implement complex algorithms in a smartphone have been pointed out by some authors, such as Zhang in [24], but a methodic study of resource consumption has not been offered.

In the review by Habib et al. [28], the authors even discouraged the use of the phone as both a sensing node and a communication gateway since these combined tasks can easily deplete the battery in no high-end smartphone models. As an alternative, they suggest the integration of external inertial units to alleviate the load in the smartphone.

Mellone et al. conceded, in [29], that the battery of the mobile phone in their testbed is depleted in less than 11 h due to the activity of the fall detection app. Mehner et al. measured, in [23], the energy demanded by a Samsung Galaxy S when running an FDS application, showing that around 8% of the battery capacity is consumed by the detection app. Tests indicated that the energy consumption does not depend on the patient‘s activity (i.e., number of falls). Reductions of 2% and 1% battery life per hour were reported by Teixeira et al. in [30] while a simple threshold-based FDS was operating in Google Nexus One, HTC Desire and Nexus S models, respectively. Likewise, after executing an FDS app in Sony Xperia U-series smart phone during a period of 7 h, the study by Kau et al. in [31] estimated that the proportion of the energy consumption due to the monitoring application is around 9% (a value similar to that caused by an interactive game). These authors remarked that this percentage can be strongly dependent on the computational complexity of the detection algorithm.

In a similar way, the neural network implemented as the core of an FDS in a HTC Incredible model is found to be the source of up to 31% of the total battery drain in the study presented by Kerdegari et al. in [32]. The app, which was tested as a background service in the absence of any other application, was responsible for limiting the battery lifespan to a single day.

The thesis by Rudraraju in [33] computes the effects of the activation of the FDS app on the time needed to deplete the battery level of the phone from 100% to 80%. By extrapolating the results, authors conclude that the lifetime of the battery is diminished by 20% because of the FDS.

A key factor that may remarkably impact on consumption is the sampling rate at which the inertial measurements are captured. In a wearable FDS system a minimum rate in the range of 25–50 Hz is required to achieve a reasonable trade-off between the battery consumption and the capacity of the system to properly characterize the body dynamics. After analyzing the influence of the sampling rate on the performance (specificity and sensitivity) of diverse fall detection algorithms, the study in [34] recommends to set a sampling frequency of 40 Hz as a good trade-off between the fall discrimination rate and energy consumption. The theoretical basis for this election can be related to the fact that the majority of the power spectrum of the acceleration signals generated by the movements of the body trunk for most daily activities concentrates in the frequency band below 20 Hz [35,36]. A similar sampling rate of 50 Hz is recommended in [37] based on some initial empirical study of the human movements.

Aiming at moderating consumption, Aguiar et al. proposed, in [38], to lessen the accelerometer sampling rate from 67 Hz to 4 Hz if the patient is detected to be motionless. Performed tests show that this technique (also suggested by Serafimov in [39]) allows cutting down the energy needed by the detection app from 2.21% (of the total capacity) per hour to only 0.19%.

The number and typology of embedded sensors that are considered by the FDS algorithm may also affect the consumption. The tests performed by the aforementioned study by Mellone in [29] reveal the correlation of the consumption and two factors: the sampling frequency and the number of embedded sensors that the smartphone employs for the detection decision. Thus, the battery lifetime of a Samsung Galaxy S II may vary from 16 h up to 30 h (for a sampling rate of 100 Hz), if all the three internal IMU sensors (magnetometer, gyroscope and accelerometer) or just one of them (accelerometer) are active. Despite the possible advantages of using other inertial or position sensors, Aguiar et al. justified, in [38], the election of the accelerometer as the unique sensor of a smartphone-only based FDS since accelerometers are proved to be more energy-efficient than the other inertial sensors. In the study of the smartphone-only based FDS presented in [40], Figuereido et al. also remarked that the energy demanded by the gyroscope is much higher than that required by the accelerometer.

In the domain of the techniques employed to process the signals and detect the falls, the abovementioned study in [40] emphasized the idea that the algorithm of the detection app may also hamper the battery autonomy. Hence, these authors recommended deploying simple threshold-based techniques instead of more complex machine-learning strategies, which are inherently more resource-demanding. Nevertheless, no experimental analysis was performed to evaluate the impact of the selected algorithm on the device lifetime. The election of simplistic threshold-based (TB) detection techniques in smartphone-based FDS is also justified in [28] as they are assumed to be less battery consuming than machine learning strategies for their lower computational costs. A similar defense of ‘thresholding’ algorithms is presented by Kostopoulos et al. in [41] to avoid more sophisticated non-parametric classification methods. Viet, in [42], proposed to use a simple acceleration peak detection of forward falls to lessen the energy consumption without sacrificing accuracy. Nevertheless, in none of these works has the consumption of the FDS been measured to assess the effects of the detection technique on the consumption. Oppositely, the work by Carletti et al. presented in [43] compared the latency as well as the effects on battery and memory usage of three well-known machine learning strategies (k-Nearest Neighbors, Support Vector Machine and Self-Organizing Maps) when they are used in smartphone-only based system to detect falls as anomalies. The memory and battery required by the algorithms, respectively, range between 28 and 40 Mb, and between 1% and more than 2% of the global energy required by the smartphone (a Huawei P8 lite model running Android 6) during 10 min.

One major factor when using machine learning strategies (especially if they are applied in a phone) is the election of the inputs that have to feed the classifier. In the smartphone-only based FDS introduced in [43], authors state that the raw signals should be preferred as input features in order to avoid computing in real time more complex statistical features derived from the measurements. However, if a high number of raw samples are employed as features, the complexity of the detector and the requirements of memory space of the algorithm can also increase.

The importance of the communication interfaces on the consumption of an FDS has been underlined in the review on wearable FDSs presented by Delahoz and Labrador in [44]. This work suggests certain strategies to diminish the energy drained by wireless communications that are required to receive the mobility data from the external sensors. Among these strategies, authors mention the data aggregation and compression (to reduce the number of transmission) or to delegate part of the detection decision on the external sensor. Yet, these authors admit that these techniques may also jeopardize the efficacy of the FDS.

Another source of consumption in the mobile phone is caused by the use of GPS. In many practical contexts of an FDS, where user monitoring is not supposed to be restricted to a certain ‘controlled’ environment, fall alarm messages should include the GPS coordinates so that the location of the fallen patient can be determined by the remote observation point. By performing systematic 6 h tests, Luque et al. showed in [27] that the constant use of the GPS sensor by the detection app significantly degrades the autonomy of the smartphone models employed in the testbed (HTC Desire X and HTC Sensation XE).

Moreover, there are even less studies that thoroughly investigated the memory and computing resources required by the detection applications. After examining the CPU computation load caused by the detection app in [45], Hou stated that the processor speed of the smartphone is not a key constraint for the deployment of FDS apps. However, the typical failures due to the multitask nature of Android platforms are normally neglected by the related literature. As far as we know, no work has been devoted to evaluating the coexistence of the conventional utilities of the smartphone (phone calling, messaging, web browsing, video playing, etc.) and a fall detection application that is constantly monitoring user movements.

As it regards the disk space requisites for hosting an FDS app, this does not seem to be a problem for the capacity of present smartphones. For example, the neural detector proposed by Kerdegari et al. in [32] just required an Android application package (apk) of 928 KB.

### 2.3. Energy Consumption in Smartphone-Based Human Activity Recognition (HAR) Systems

FDSs can be observed as a very particular example of both health condition monitoring and Human Activity Recognition (HAR) systems. In this area, some papers have addressed the problem of battery depletion in smartphone-based HAR systems.

In the review about smartphone-based activity recognitions systems presented by Shoaib et al. in [21], authors pointed out that most studies in the literature do not present any analysis about the resource consumption (mainly CPU, battery and memory usage). This review does not consider the papers focused on fall detection. The comparison of different smartphone-based HAR systems existing in the literature and presented in [46] shows that the battery lifetime of the analyzed prototypes spans from 7 to 18 h.

It has been pointed out that current mobile devices must be modified to enable the execution of applications intended for movement recognition [47] in an efficient manner. Smartphones are not designed to process the data that are continuously generated by human movements. For example, the sensor in some still existing low-end Android is not completely operative provided that the processor stays in a sleep mode, even if a background monitoring application is running. In those cases, the application developers of an FDS are forced to set a “wake lock” to avoid the processor moving into low consumption mode at the expense of a dramatic increase of the power usage.

The use of resources of the smartphone in smartphone-based HAR systems has been specifically revised by Del Rosario et al. in [48]. These authors highlight the importance of the configuration (in particular, resolution and sampling rate) of the embedded sensors when determining the depletion of the battery. However, this study does not investigate the consumption caused by Bluetooth connections when external sensors are incorporated to the HAR architecture.

The thesis by Viet in [49] analyzed the energy depletion rate in a smartphone employed in a mobility recognition system. The study evaluates the influence of the sampling frequency on the energy expenditure, showing that a compromise between the efficacy of the detection accuracy and the consumption is always required.

In [50], a ‘hardware-friendly’ approach is followed to implement an smartphone-only based prototype aimed at HAR purposes. In particular, the standard Support Vector Machine (SVM) classification method is adapted to exploiting fixed-point arithmetic in order to reduce the computational cost. Some tests of this proposal are presented in [51], where the results indicate that the election of fixed-point arithmetic significantly smooths the battery drain rate of a Samsung smartphone. A similar approach is followed by Alvarez et al. in [46] to develop a HAR and FDS. In this work, a lower battery consumption is achieved by using discrete variables instead of continuous ones.

### 2.4. Consumption in Hybrid Fall Detection Architectures (Combining External Sensors and a Smartphone)

Several hybrid architectures (i.e., those combining a smartphone and one or several external additional sensors) have been proposed by the literature. In some cases, the smartphone operates under a more ambitious monitoring scheme not only intended to detect falls but also other biosignals. For example, the systems presented by Horta et al. in [52,53] integrate a smartphone and a set of Bluetooth-enabled medical sensors aimed at providing biofeedback monitoring. In other cases, the additional sensors are incorporated to the FDS in order to help the personal device with the detection decision. Thus, the combination of a smartphone and an external accelerometer to detect falls is utilized in the prototypes presented in [45,54,55,56,57].

In a hybrid FDS, the design of the interaction between the smartphone and the external sensors to generate the detection alarm may follow three different strategies [20].

Firstly, according to a policy of completely local processing, the external sensor would be in charge of analyzing the captured signals and producing the binary detection decision. In this case, the smartphone only operates as a simple gateway that retransmits the decision of the external sensor (via HTTP Push notifications, SMS, phone calls, etc.) to the remote center where alarms are received. The main drawback of this approximation is that the heavy hardware restrictions (i.e., memory and computing power) on the wearables do not usually allow implementing complex (and more accurate) detection algorithms. Moreover, the features and classifiers used in detection applications proposed by the literature are normally designed just to achieve a high accuracy, while disregarding the impact of the detection algorithm on the resources of the sensing motes. Thus, with independence of their actual efficacy to discriminate falls, in many cases, they cannot be actually deployed on an actual low power external wearable units [58]. In this vein, the authors in [59] highlight that the use of windowing techniques and complex mathematical operations cannot be executed in sensing boards with a small amount of RAM or with an MCU that does not support floating point arithmetic. Yet, the increasing computing capacities of wearable technologies and the optimization of deep learning architectures used for fall detection may be changing this panorama. For example, Torti, Fontanella et al. have shown, in [60], that an FDS based on a Recurrent Neural Network (RNN) can be implemented on a STMicroelectronics SensorTile platform that embeds a micro controller unit, two tri-axial accelerometer, a gyroscope and a magnetometer. In particular, the authors analytically prove that the running detection algorithm (which achieves a good classification accuracy when it is tested against a public repository of mobility samples of ADLs and falls) can run on the mote for more than 130 h without recharging. In [61], Torti, Musci et al. extend the study of this FDS to assess the computational costs and the battery consumption that are required when the SensorTile board employs Bluetooth Low Energy to inform about the status of the user. The authors conclude that the impact of BLE communications is negligible if Bluetooth communications are only utilized when specific events (e.g., a suspected fall) take place. A similar detector, also based on a RNN, is also presented by Luna-Perejón in [62]. The FDS (which intercommunicates with a smartphone via Bluetooth) is implemented on a board that integrates an F411RE microcontroller and an ADXL345 accelerometer. The lifetime of the 150 mAh battery that powers the wearable module is estimated to be between 38 and 53 h, depending of the number of transmitted alarms. In any case, commercial off-the-shelf motes employed for these prototypes only embed short-range low-power connection technologies, so an external gateway must be located in the close vicinity of the mote to receive the alarms, which reduces the freedom of movements of the monitored user. In the studies where the mote is provided with a wireless interface with a higher transmission range, such as Wi-Fi in [63,64], GSM in [65,66,67,68] or both GSM and wi-fi [69], the battery lifetime of the system is not usually evaluated.

The second alternative is to avoid this computational load in the external sensor by transferring all the system intelligence to the smartphone (which, in certain architectures for home-automation environments, could be replaced by a fixed base unit). Under this scheme, the sensor is forced to constantly transmit the inertial measurements to the smartphone, which can deplete the batteries of both the sensor and the phone due to the wireless communications.

A third alternative is to execute some kind of basic preprocessing in the wearable so that the signals (e.g., the IMU measurements captured for a fixed time window) are only transmitted when a certain event is detected (e.g., the acceleration magnitude exceeds a certain threshold) and thus a fall is suspected. The final decision in that case is taken in the smartphone (or other external intelligent gateway), where a more sophisticated detection technique is implemented to analyze the received signals. This strategy (also recommended by Serafimov in [39] as a key technique to cut down the consumption) is followed by Casilari in [70] and by Blasco in [71]. However, in both cases, the benefits of this two-phase cooperative detection (with respect to the case in which no preprocessing takes place in the sensing node) are not investigated.

Due to the high consumption of Wi-Fi interfaces, there are very few studies in the literature on wearable FDS (such as that by Hyunh et al. in [72] ) that have considered the use of this LAN standard to deploy the communications between the external sensor and the decision core of the system. In most cases of hybrid architectures, external sensors are equipped with low-power and low-consumption technologies, which are more appropriate to guarantee a longer battery life. In this context, although ZigBee [71,73,74,75] or RF (Radio-frequency) [13] have been selected as the low-power low-range transmission technology in certain proposals of (not smartphone-based) FDS in the literature, Bluetooth Low Energy is normally preferred, as far as it is natively integrated in most present smartphones and wearables units conceived to track the human mobility.

In this context, Bluetooth 2.0 is employed in the hybrid prototype presented in [58] but authors state that Bluetooth 2.0 connections deplete the 600 mAh battery of the external mote in less than 4 h. In [45], Hou et al. estimate a battery lifetime of only 7 h in a HTC terminal when the mobile is utilized to receive the data via Bluetooth from an external accelerometer and decide if a fall has occurred. Majumder et al. present an architecture [76] that integrates a smartphone (an iPhone model) and a smartshoe (containing four pressure sensors) that intercommunicate via Wi-Fi. The tests applied to the iPhone model reveal that the detection application reduces the battery lifespan to less than 3 h.

Another interesting hybrid wearable solution is the architecture that combines a smartwatch and a smartphone. In these cases (such as those presented in [77] or [26]), the two devices also communicate via Bluetooth. However, despite their widespread use for personal monitoring during sporting activities, the low capacity battery and small screens of smartwatches are claimed to be two noteworthy barriers for the adoption of this wearable technology in the area of health monitoring of elderly people [78]. In fact, most existing smartwatches suffer from heavier restrictions than smartphones in terms of computing power and energy autonomy. Studies of FDS that employ a smartwatch have shown that the FDS application can result in a severe decrease of the lifespan of the smartwatch battery [70,79].

The election of the sampling rate is even more critical for a smartwatch than for a smartphone to minimize energy depletion. Authors in [77] show that using the lowest possible accelerometer sampling rate may extend the usage of the smartwatch (a Pebble model) of the proposed FDS beyond 30 h.

The consumption induced by an Android acceleration-based fall detection app is analyzed by Dai et al. in [80]. The proposed system also includes a magnetic sensor to enrich the decision algorithm. However, the authors do not clarify if this ‘magnetic accessory’ really connects to the smartphone. Conducted tests show that the app provokes an extra reduction of 10% of the total battery level after 6 h of monitoring.

In any case, the analysis of the related literature reveals a lack of a systematic study of the consumption of battery and hardware resources in a smartphone when they are operating as the core of a wireless network of inertial sensors aimed at detecting falls. In this field, no study has addressed the impact of other high resource consuming apps (e.g., those for multimedia decoding) on the performance of a fall detecting app.

## 3. Description of the Experimental Testbed

The evaluation testbed consists of a Body Sensor Network (BSN) comprising a smartphone (acting as the gateway and the central node of the architecture) and a variable number of Bluetooth-enabled wearable motes that incorporate an IMU. In this section, we describe the characteristics of the smartphones, the developed mobile application and the sensors employed to perform the measurements.

### 3.1. Employed Smartphones

Four different smartphones have been employed to perform the evaluation of the battery durability. The employed models were chosen to cover a wide and representative variety of vendors and versions of the Android Operating Systems. In our tests, we only consider Android devices as long as Android is indisputably the most widespread Operating System (OS) in the smartphone market, with an 87.0% share and a shipment volume of 1193.2 million units during 2019 [81]. Future research should extend the analysis to iOS and other alternative operating systems.

The main characteristics of the employed smartphones are summarized in Table 1.

These smartphones (which include models of two of the three major vendors of Android technology [81]) were selected to examine the influence of Android and Bluetooth version. As can be seen from Table 1, Android versions from 6.0 (Marshmallow) to the most recent 9.0 (Pie) release are represented. As of July 2019, these versions account for more than 84% of the existing Android phones [82]. Similarly, the testbed devices incorporate the different revisions of the Bluetooth 4 standard (4.0, 4.1 and 4.2) as well as the latest 5.0 version (implemented on the Xiaomi Mi A2). Moreover, the four smartphone models present an analogous battery capacity (around 3000 mAh) except for the Samsung Galaxy S6, which has a capacity of 2550 mAh.

### 3.2. Mobile Application

In order to appraise the battery drain during the operation of the body sensor network with the wearables, a mobile application (app) has been developed. This app is in charge of discovering the sensors in the vicinity, setting up the corresponding Bluetooth connections to them and receiving and processing the IMU signals captured and transmitted by the motes. The app allows configuring the following parameters and operations:

- The number of sensors (up to 6) that form the network and are allowed to connect to the phone.
- The use of the GPS information. The app permits to decide if the coordinates of the smartphone will be collected (or not) by using the embedded GPS sensor. This option enables to evaluate the impact of the GPS localization on the battery drain as the GPS service is programmed to be operative during the experiments.
- The use of the smartphone as a gateway to constantly retransmit the signals. In an actual detection application, the smartphone could be used in two different ways: as a fall detector or as a simple gateway that retransmits the information captured by the sensors. In the first case (if the fall detection algorithm is locally implemented in the phone), only the detection alarms are supposed to be transmitted to the remote monitoring point. On the contrary, if the fall detection algorithm is ‘outsourced’ to an external server in the cloud (for example, to avoid the limited computing resources of the smartphone), the sensors’ measurements (the three components captured by the triaxial gyroscope, accelerometer and magnetometer) have to be permanently conveyed in real-time to the Internet. This option requires a Wi-Fi or a 4G connection to be operative and, consequently, will also increase the battery drain.
- The sampling rate of the sensors. The app enables setting the sampling period of the sensors from 10 ms (100 Hz frequency) to 2550 ms (0.39 Hz), which are the maximum and minimum values with which the sensors can be adjusted.

In the design of the app, the user interface has been minimalized to reduce to a minimum its influence on the consumption. In particular, the text data (indicating the last received data) shown for feedback purposes are updated only after every ten seconds. Moreover, every two minutes, the app stores the following information in a log or trace file:

- A timestamp.
- The battery level.
- The current battery voltage and the instant current that is being consumed.
- The smartphone temperature.
- The cumulative number of received and lost messages from each sensor (since the start of the experiment).

In addition to this regular logging, as soon as the app detects that the smartphone is being turned off because the battery is almost depleted, it stores the final data collected and close the trace file. The resulting log files have been processed through Matlab scripts [83].

### 3.3. Sensors

Up to six Texas Instruments CC2650 SensorTag motes have been used in this study as the BSN mobility sensors [84]. The CC2650 SensorTag is a Bluetooth-enabled tiny battery-operated module that incorporates an MPU9250 IMU from InvenSense. This IMU, in turn, embeds three tri-axial sensors: a gyroscope, an accelerometer and a magnetometer. The accelerometer range can be configured with four different values: 2g, 4g, 8g and 16g. We selected a range of 4g as it is normally enough for fall detection (in any case, the selection of this value does not affect the consumption). In addition, the SensorTag motes integrate other three extra sensors: a HDC1000 humidity sensor from Texas Instruments, a BMP280 barometric sensor from Bosch Sensortec, and an optical sensor OPT3001 from Texas Instruments.

By default, the minimum sampling rate that is defined by the SensorTag module to access the IMU sensors is 100 ms (10 Hz). As commented in the previous Section 2.2, this frequency cannot be sufficient to characterize human mobility in a fall detection system. This limit of the sampling period is hard coded in the SensorTag firmware and hence, it had to be modified to be 10 ms (100 Hz), which is the minimum resolution allowed by the sensors. Additionally, the firmware was also modified in order to disable the humidity, barometric pressure and optical sensors in the modules as they were not going to be utilized in the experiments. This releases the processor from the corresponding sensing tasks, increasing its capability to process the IMU data at a rate higher than originally expected.

By means of a Bluetooth connection, each SensorTag periodically sends to the application running in the smartphone the messages with the data received from the inertial sensors. In the software provided by the vendor, there is not a procedure to keep track of the messages that have been lost. In this light, the SensorTag firmware was also modified to add a sequence number to every message sent to the app. In this way, the app can implement a simple count mechanism to identify lost packets.

### 3.4. Performance Metrics

We have executed a battery durability test for all the designed configurations of the testbed. The general testing method was to fully charge the smartphone battery and configure the app to connect to a predetermined number of sensing nodes with some predefined parameters. Once the corresponding Bluetooth connections are established, the app starts receiving the inertial data from the SensorTags and the transmission continues until the smartphone battery is depleted. Since the goal was to quantify the energy drain in the phone, the SensorTags were USB-powered instead of being battery operated (with CR2032 coin cell batteries). This prevents malfunctions or interruptions in the wireless transmissions caused by energy shortage problems of the external nodes.

The analysis has been mainly focused on two factors: the election of the sampling rate and the number of employed sensing nodes. Thus, the analyzed scenarios are as follows: firstly, the influence of the sampling rate on the battery consumption has been investigated. Then, a sampling period of 20 ms (50 Hz) was fixed in order to study the influence of the number of external nodes. In both cases, we also assessed the impact of activating (or not) the smartphone’s GPS sensor as well as the role assigned to the phone in the architecture: 1) as a complete FDS that would eventually inform only about fall occurrences or 2) as a gateway that retransmits the IMU measurements to a remote server.

The performance metrics employed in our study are:

- Battery duration (in hours), measured as the time elapsed since the beginning of the experiment (in which the battery is fully charged) until the moment in which the mobile turns off due to the battery discharge.
- Relative battery duration (expressed in s/mAh), defined as the ratio between the battery duration and the battery capacity. As the battery capacity of the employed smartphones are not exactly the same, this metric characterizes the lifespan of the batteries when the same energy is provided.
- Number of received message per consumed energy unit (messages/mAh). It is defined as the total number of notification messages sent by the sensing modules and received by the smartphone divided by the battery capacity. This metric describes the energetic cost of transmitting the measurements.
- Number of lost messages per consumed energy unit (lost messages/mAh), computed as the ratio between the total number of notification messages lost in the transmission from the sensors to the smartphone and the battery capacity. This parameter characterizes the losses experienced by the system during the tests.

## 4. Results and Discussion

### 4.1. Influence of the Sampling Rate

**Figure 1:**
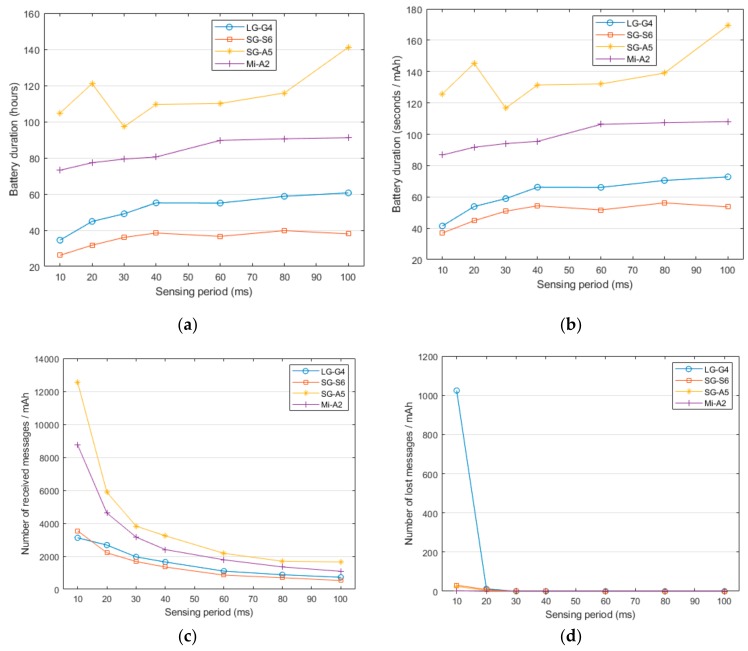
Performance metrics as a function of the sampling period. (**a**) Battery duration; (**b**) relative battery lifetime (lifespan per consumed mAh); (**c**) number of messages received per consumed mAh; (**d**) number of lost messages per consumed mAh.

In this section, we study the influence of the sampling frequency of the sensing period on the battery depletion. To this end, we repeated the durability test by varying the sampling periods from 10 ms (100 Hz), which is the maximum sensing frequency of the SensorTag, and 100 ms (10 Hz). All the results, which are represented in Figure 1, were obtained for the case in which the BAN employs a single sensing node (i.e., one SensorTag).

Figure 1a shows that the battery duration strongly depends on both the smartphone model and the employed sampling period. As can be observed, the smartphones running the oldest Android versions (LG-G4 and Samsung Galaxy S6 models) present the lowest battery lifetime as well as a major dependence on the selected sampling periods. In particular, the use of the highest data rate (100 Hz) provokes a reduction of the battery duration of these models of up to 50% compared with the cases under the lowest sampling rate. A similar conclusion is derived from the examination of the curve describing the relative lifetime (Figure 1b). On the other hand, the consumption in the Samsung Galaxy A5 and Xiaomi Mi A2 models is more moderate and exhibits a much smoother evolution as the lifetime shows a more subdued increase (of up to 30%) when the sampling period diminishes.

Figure 1c, in turn, represents the number of messages received by the smartphones per mAh as a function of the sensing period. It follows a complementary behavior to that represented in Figure 1a,b as the lower the sampling period, the higher the number of received messages is. The figure illustrates that the energy cost of transmitting a message strongly differs depending on the model. Thus, the LG-G4 and Samsung S6 process less messages than the Xiaomi Mi-A2 and Samsung Galaxy A2 if we consider the same consumed energy. However, this difference is diminished as the sampling period increases.

**Figure 2:**
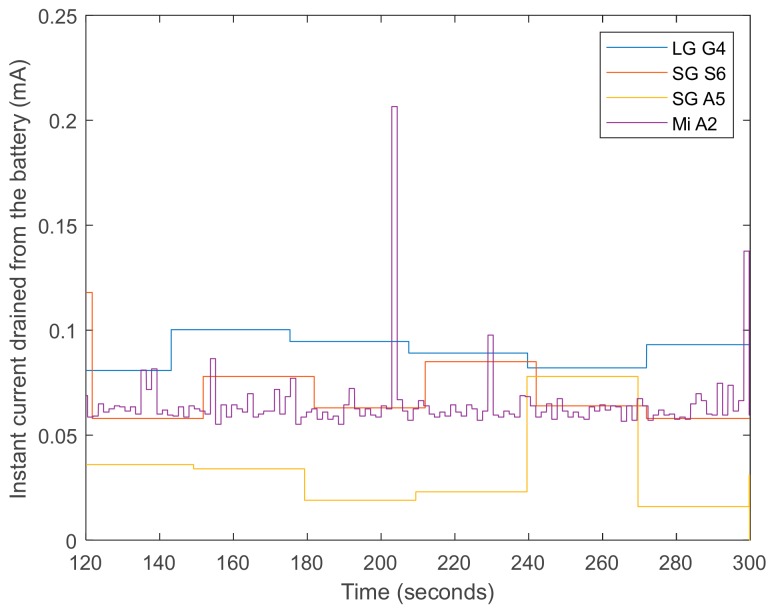
Snapshot of the evolution of the current drained for the four analyzed models for three particular minutes (from second 120 to 300) of the experiment with a frequency sample of 50 Hz and one connected sensing node.

In general, the results seem to confirm that the use of the battery has been progressively optimized by the successive versions of Android. Figure 2, which depicts the evolution of the current drained from the smartphone battery while the app is running (for the same three minutes—3 to 5—of the experiments), shows that the dynamics of the energy depletion in the four models follows quite different patterns. In fact, Android 8 introduced some policies to optimize the battery life [85], which were improved for version 9. However, the battery manager can also interfere with the normal operation of a background application that is constantly making use of the networking capacities of the smartphone. As illustrated in Figure 1a, the Galaxy A5 model, which provides the longest battery life, may present an anomalous or inconsistent behavior for low values of the sampling period (i.e., for high data rates). From a detailed study of this model, we concluded that the operating system (Android 8) in the Galaxy A5 is programmed to drastically reduce the battery consumption by stopping the applications that have been running for a relative long time. Figure 1d represents the number of lost messages as a function of the sampling frequency. This figure shows that only the LG G4 smartphone presents serious problems when using the highest sampling rate. This point is confirmed if we examine, in Table 2, the global packet loss ratio that is achieved by the four models for a sampling period of 10 ms (100 Hz). The table reveals the difficulties of the least recent model (LG G4) to receive the measurements without losses for this frequency of 100 Hz. In particular, the losses suffered by the LG G4 model (for a sampling rate of 100 Hz) reach 24.7% of the total generated Bluetooth messages. This indicates that some models have problems operating with a high data rate. Accordingly, for the rest of the experiments in the following sections, we set the sampling rate to 50 Hz (sensing period of 20 ms), a value which simultaneously fulfills the requisites for an adequate sampling rate in an FDS and minimizes the transmission losses.

In any case, as illustrated by Figure 1a, the lifetimes range from approximately 27 h for the worst case (Samsung G6 with the highest sampling rate—100 Hz) to 141 h (Samsung A5 with the lowest sampling rate—100 Hz). This implies that all the studied models remain active after 24 h of operation even if a high sensing rate is selected. Hence, in a realistic scenario and for all models, the battery could guarantee that the user is permanently monitored during his/her daily routines without needing any recharge until the bedtime.

### 4.2. Influence of the Number of Sensors

In this experiment, we assess the performance of the smartphones when modifying the number of sensors that are wirelessly connected to them. Thus, for a sampling period of 20 ms (50 Hz), we compare the battery consumption when up to six SensorTags are simultaneously sending their measurements via Bluetooth to the smartphone, which act as the central node (master) of the Bluetooth piconet.

**Figure 3:**
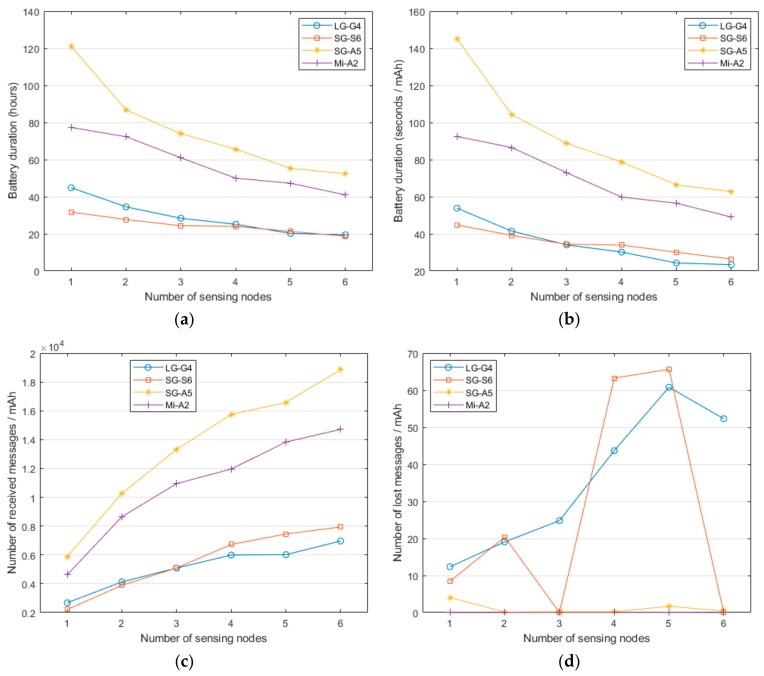
Performance metrics as a function of the number of connected sensors for a sampling rate of 50 Hz: (**a**) Battery duration; (**b**) relative battery lifetime (lifespan per consumed mAh); (**c**) number of messages received per consumed mAh; (**d**) number of lost messages per consumed mAh.

As the number of connected sensors grows, the battery durability is expected to decline due to the increasing number of wireless transmissions and messages that have to be processed by the operating system. The analysis of Figure 3a,b, which show the battery duration (in absolute terms and for the same consumed energy) as a function of the number of employed SensorTags, endorses this prevision. Again, the Samsung Galaxy A5 (Android 8) exhibits the lowest current consumption, followed by the Xiaomi Mi-A2 (Android 9), Samsung Galaxy S6 (Android 7) and LG-G4 (Android 6). It should be noticed that this decrease of the lifetime experiences a similar decay rate for all the models except for the case of the LG-G4, whose energy drain rate seems to grow slightly faster as the number of sensors augments. In any case, the graphs reveal that the network dimension unmistakably impacts on the battery lifetime although this reduction is not linearly dependent on the number of sensors.

Thus, the battery lifetime of the system with six sensing nodes is approximately halved with respect to the reference network with just one SensorTag. This implies that with two of the models and the highest number of motes, the battery is not expected to live longer than 20 h, which could hinder its utilization in a realistic monitoring application.

These trends are confirmed if we get an insight of the energy cost of transmitting messages (see Figure 3c). Moreover, from some preliminary tests, we observed that the Samsung Galaxy A5 presented significant issues to simultaneously manage more than two Bluetooth connections with the nodes, as long as the number of lost messages skyrocketed when three or more SensorTags were integrated into the BSN. As already mentioned, during the development of the app, we verified that the Samsung Galaxy A5 (Android 8) is the most aggressive one with respect to the battery management as it directly ‘kills’ or restricts those background applications that the operating system considers as battery drainers. Hence, this model closes the Bluetooth connections by killing the corresponding application thread. To overcome this drawback, we had to develop a reconnection mechanism in the app that reopens the Bluetooth communications when this disconnection is detected. Additionally, the Bluetooth system service had to be configured to be unmonitored by the operating system in order to prevent the Bluetooth connections from being closed. Consequently, only the LG G4 and Samsung Galaxy S6 smartphones, those with the lowest capacity, lost some messages. Table 3 summarizes the percentage of lost messages, showing that (in any case) they are lower than 1%, which can be considered as an acceptable loss rate for an application intended for a constant user monitoring.

### 4.3. Influence of the Use of Localization Services

Geolocation is a convenient feature of a wearable FDS (especially if the user loses consciousness after a collapse) as it allows to inform the remote monitoring point (e.g., the family or an assistance provider) about the user’s location as soon as a fall is detected. In this section, we extend the previous analysis to measure the consumption in the smartphone caused by the monitoring app when the positioning system (GPS) is activated. In this regard, the Android Application Programming Interface (API) enables configuring the requests to the GPS sensor on a periodic basis (according to a fixed preset interval) or, otherwise, when the displacement of the Android device with respect to the last obtained position has exceeded a certain distance. As the smartphones were static during the experiments, the app was programmed to request the GPS position every ten seconds in order to simulate a continuous movement of the user (which would be the most demanding scenario in terms of the energy required by the location service).

**Figure 4:**
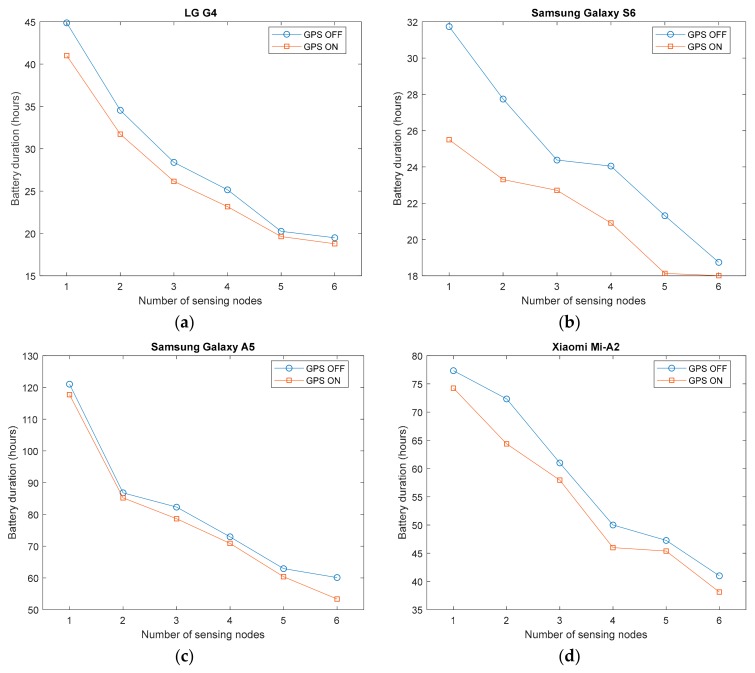
Battery duration as a function of the number of connected sensors for a sampling period of 20 ms (sampling rate of 50 Hz) depending on the use of the localization services for the different smartphones: (**a**) LG G4; (**b**) Samsung Galaxy S6; (**c**) Samsung Galaxy A5; (**d**) Xiaomi Mi-A2.

Figure 4 depicts the evolution of the lifespan of the battery as a function of the number of sensors depending if the GPS is activated or deactivated during the monitoring.

Although the energy drained by the GPS module may rely on other factors, such as the SNR of the signals received from the satellites [86], the four graphs in Figure 3, which separately display the behavior for each smartphone model, show that the permanent GPS readings of the app may notably reduce the battery lifetime (around 5–10% of the total duration in all models except for the case of the Samsung Galaxy S6, for which this reduction even reaches up to 20%). This not negligible impact on the battery depletion caused by a GPS-dependent app could be minimized if the GPS receiver is only turned on as soon as a fall is suspected. However, in that case, the response time of the fall alert could be affected by the long startup delay that is required by the GPS chip to synchronize with the GPS satellites.

### 4.4. Influence of Using an External Server

As previously commented, a smartphone in an FDS can be used as a simple gateway only in charge of retransmitting the measurements collected by the inertial sensors to a server in the Internet cloud. Thus, instead of locally implementing the intelligence of the FDS on the phone, the external server will process the signals and generate the alarm if a fall is identified. The use of this server may ease the exploitation of more sophisticated detection algorithms (which cannot be deployed on the limited resources of a smartphone) as well enabling the continuous tracking of the user’s movements (and not only the reception of fall alarms) at the remote monitoring point. However, this is achieved at the cost of a higher activity of the networking interfaces in the smartphone. In particular, this functionality requires a wireless connection (Wi-Fi or 3G/4G) to be operative and, consequently, it will deplete battery much faster as these interfaces are a leading component (after the use of the screen) in the energy budget of a smartphone [87,88,89].

To evaluate the consequences of implementing this new role on the phone, we repeated the previous tests but now forcing the smartphone to retransmit all the signals received from the inertial sensors (via Bluetooth) to an external Internet node through a Wi-Fi connection (a higher consumption—and a higher delay—should be expected if a 3G/4G connection had been considered instead [90]). For this purpose, in order to receive the data through TCP/IP sockets, a data server is programmed on a computer connected to the same Wi-Fi access point to which the smartphone is associated.

In order to optimize the battery usage, Android Operating System is scheduled to disable the Wi-Fi connections after a certain period in which the screen is switched off. To avoid this policy, the app was forced to keep the screen turned on (with the brightness dimmed to the minimum) during the whole experiment. Aiming at computing the additional consumption introduced by this element, we also measured the battery lifetime in the basic case when the app just activates the screen (with the same minimum brightness level) and no wireless connection (not Bluetooth nor Wi-Fi) is established. By making so, we calculated the minimum current drain required by the app (mainly caused by the screen) when no transmission takes place.

**Figure 5:**
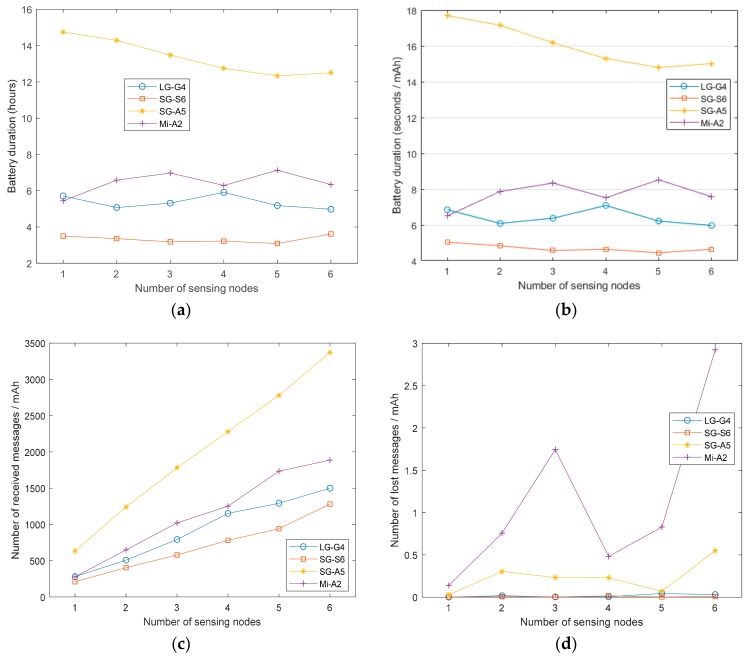
Performance metrics as a function of the number of connected sensors for a sampling rate of 50 Hz when Wi-Fi is enabled to retransmit the received data to an external server: (**a**) Battery duration; (**b**) relative battery lifetime (lifespan per consumed mAh); (**c**) number of messages received per consumed mAh; (**d**) number of lost messages per consumed mAh.

Figure 5 shows the results for this new scenario, again for the case in which the sampling rate is 50 Hz and when the number of connected sensing nodes is varied from 1 to 6. 

All the graphs in this figure clearly reveal the noteworthy impact of the retransmission on the energy drain. The current required to power the Wi-Fi connection is higher than that demanded by the Bluetooth connections to receive the same data from the sensors (even when six sensors are used) and therefore, the measured battery lifetimes plummet. In fact, the relative impact on the energy usage caused by the number of utilized sensing motes is decreased (and almost superfluous in some cases) as far as now energy depletion is completely dominated by the activity of the Wi-Fi interface.

**Figure 6:**
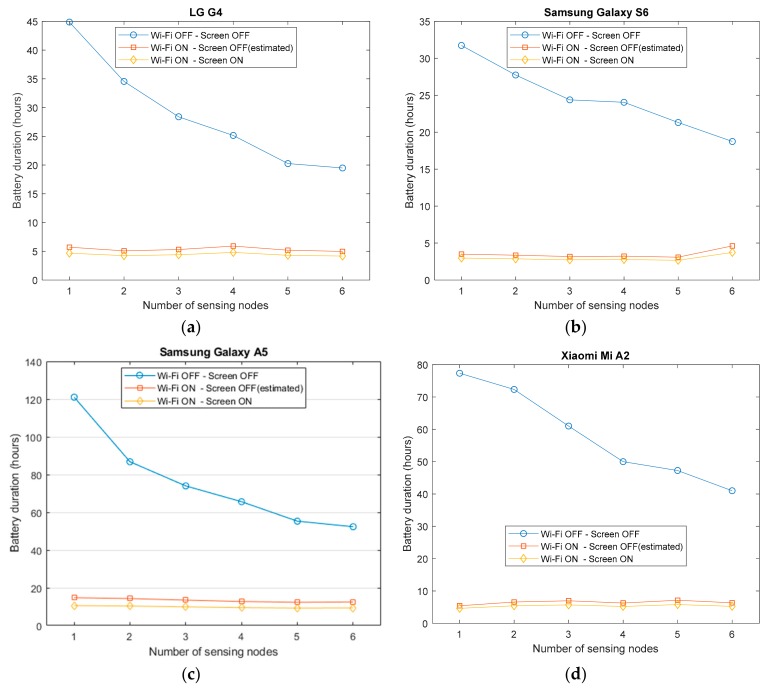
Impact of the use of a Wi-Fi connection to retransmit the received data to an external server. Performance metrics as a function of the number of connected sensors for a sampling rate of 50 Hz for the four considered models: (**a**) LG G4; (**b**) Samsung Galaxy S6; (**c**) Samsung Galaxy A5; (**d**) Xiaomi Mi-A2.

Figure 6 recapitulates the data of Figure 3a and Figure 5a and directly compares the lifespan of the battery for the cases in which the Wi-Fi retransmissions are utilized or not. The figure also represents the expected battery lifetime that would be achieved if we just take into consideration the energy drained by the normal operation of the app (subtracting from the obtained measurements the basis consumption caused by the activation of the screen, which, as commented, was previously empirically estimated). The numerical results of the graphs undoubtedly indicate that in the experiments, the screen (under the minimum programmable brightness) has a minor impact on the measurements.

We can observe that when Wi-Fi is activated, the lifetime decreases by a factor that ranges between 5 and 15, depending on the scenario (smartphone model and number of used sensors). As a consequence, the battery is expected to last between 4 and 15 h, which implies that in a realistic scenario of an FDS, the user would be forced to recharge the phone before going to bed. These results evince that current smartphones are not still capable of acting as a ‘pure’ sensor gateway in a monitoring system for fall detection. In order to enable the participation of an external server in the fall detection, some preprocessing and filtering of the data received by the sensor must be mandatorily accomplished in the smartphones. Thus, the phone should retransmit to the server not all the measurements but only those signal sequences that are ‘suspicious’ of containing a fall event (e.g., a certain time interval after and before a peak in the acceleration module that surpasses a predefined threshold).

## 5. Coexistence with Other Applications

When using a smartphone as a part of a fall detection system, we cannot forget that the implementation of an FDS should not prevent the typical use of the rest of conventional services provided by this popular personal device. Consequently, the activity of the fall detection app must coexist with other applications without disrupting their normal operation.

In order to appraise the interference between our app and other programs residing in the phone, we developed a test of coexistence with the YouTube application [91]. We selected this application because of its popularity and because it is one of the applications with the highest requirements in terms of computing resources (as it has to decode video and audio streams in real time), memory (as far as it needs a big memory space to store the data stream to be decoded) and network resources (as it continuously receives the multimedia data from the cellular or the Wi-Fi interface). In any case, similar results were obtained with other typical apps that make use of the standard functionalities of the smartphone.

Thus, we carried out a new experiment in which our app was connected to a single sensing module using a reception period of 20 ms (50 Hz) while the YouTube application was simultaneously downloading and playing a certain high quality video of 314 s [92] in full screen mode. The video playback quality was configured to be 1080p and the monitoring operation was executed during five minutes.

In order to verify the correct coexistence of both applications, we observed the video sequence and visually checked if there was any type of deceleration, glitch or subjective loss of quality of the video or audio playback. Additionally, we evaluated if the FDS app was properly receiving all the sensed data, without loss of messages and at the correct rate.

The test was repeated for the same four smartphones used in the previous sections. In all the performed experiments, the video streaming worked correctly, that is to say, without any type of visually noticeable degradation. Table 4 shows the number of received and lost messages during the test.

It can be observed that the number of received messages is almost the same in all the smartphones and close to the theoretical number of messages that must be received during an execution time of almost five minutes (the app requires just over two seconds to initialize and open the Bluetooth connections with the sensors and start receiving data, that is why the total number of transmitted messages is not exactly 15000, which would correspond to the total amount of packets generated at a rate of 50 Hz for a period of 300 s). Table 4 confirms that the percentage of lost messages is almost zero for all the tested devices, which indicates that the monitoring application is not affected by the simultaneous activity of another resource demanding app.

## 6. Conclusions

Smartphones have been proposed as a cost-effective device to program and deploy wearable fall detection systems. Although current smartphones natively embed inertial sensors that can be leveraged to characterize the human movements, it cannot be expected that, in a real application scenario, the user to be monitored transports the smartphone in a fixed position and tightly attached to the body. To avoid an unnatural placement and use of the smartphone, the FDS can be enriched with external sensors so that the sensing function can be transferred to a set of small external IMUs, which can be transported on a certain part of the body in a more ergonomic way. 

This paper has empirically assessed the actual capability of current smartphones to perform as the central node of Body Sensor Network aimed at detecting falls. In particular, our research has focused on the impact of the wireless connections to the external sensors may have upon the battery drain, but also upon the stability and the operability of the phone. For that purpose, we configured a testbed consisting of a simple star network in which up to six low-power sensing nodes (which could be placed on different points of the body) communicate via Bluetooth with a commercial smartphone to transmit the measurements captured by three tri-axial sensors: an accelerometer, a gyroscope and a magnetometer. 

The study thoroughly and individually analysed the influence of several parameters that may affect the consumption: the sampling period at which measurements are taken and transmitted, the number of sensors in the BSN, the use of a positioning system and the role of the phone in the FDS (as the decision core that only informs about falls, or as the hub or gateway that simply retransmits to an external server the signals received by the sensors).

In our testbed, four different smartphone models of popular vendors with different Android versions (ranging from Android 6 to 9) have been employed. In addition, a mobile application has been developed in order to keep track of the battery duration.

As could be expected, the sampling rate and the number of sensing nodes clearly influence the achieved battery lifetime. In the case in which the smartphone is not used as a simple gateway, the energy required by Bluetooth connections when six sensors are simultaneously operating may halve the battery duration when comparing with the basic case in which just a single sensor is considered. Similarly, a high sampling rate of 100 Hz can multiply the current drain by a factor of 6 with respect to the case in which a much lower sampling frequency (10 Hz) is defined. In any case, the obtained results indicate that even for the least recent models, the smartphone can operate for a period longer than 24 h without requiring recharge if just one or two sensing nodes and a sampling rate not higher than 50 Hz are utilized. As the related literature shows that these conditions (a sampling rate about 50 Hz and one or two inertial sensors) are normally enough to characterize and discriminate the movements caused by a fall, our results imply that most current commercial smartphones could perform as the ‘sink’ or central node of a medical BSN intended for fall detection. Moreover, by testing the coexistence of the detection app in the mobile with other power-hungry applications (such as the downloading and reproduction of Internet video clips), we have shown that the activity of FDS does not interfere with the quality perceived by other services provided by the smartphone.

Concerning the Android version, which determines the implemented version of Bluetooth, the experiments reveals that the most recent models optimise the battery consumption. However, as it has been noticed for the phone implementing the Android 8 version, the operating systems may manifest some unstable or undesired behaviour under heavy traffic load (e.g., when a high sampling rate is set or when several sensors are simultaneously transmitting packets). This spurious behaviour is caused by the battery management of the Android OS as the version 8 is programmed to close the Bluetooth connections as soon as it perceives a high battery consumption. Obviously, this proactive policy would increase the battery duration but at the cost of a high loss rate of the data sent from the sensors. In our architecture, this problem was overcome by configuring the Android operating system to not monitor the Bluetooth service as well as by developing a reconnection mechanism that comes into operation whenever a Bluetooth disconnection is detected.

Moreover, after a study of the influence of the use of the positioning system on the FDS, we have shown that the effect of the operation of the GPS sensor on the battery depletion is not negligible. When this sensor is activated, the battery duration is estimated to decrease by a percentage between 10% and 20% depending on the smartphone model and the configuration of the BSN.

Finally, we investigated the power consumption of the networking architecture in the case when the fall detection algorithm is supposed to be ‘outsourced’ to an external server that must receive all the measurements captured by the wearable sensors. In this situation, in which the smartphone works as a direct gateway between the sensing nodes and the server, we have verified that the use of a Wi-Fi connection to retransmit the sensed data to the server dramatically drains the smartphone battery, reducing its lifetime to some hours. This short lifetime (which would require recharging the device several times a day) renders unpractical the use of the phone as the core of the BSN under these circumstances.

Further studies should contemplate repeating these tests with another models and smartphones with other operating systems (mainly iOS). In addition, the analysis should be extended to assess the effect on the consumption of some particular Android elements, such as the personalization layer of the vendor, or the use of the cellular data connectivity (instead of Wi-Fi) in the phone to retransmit the data to the server. In addition, the feasibility of implementing the different families of fall detection algorithms proposed by the literature should be investigated in detail. This work has primarily focused on the energy drained by the wireless connection. However, in spite of the increasing computing capacity of current smartphones, the implementation and execution of some complex algorithms can still pose a challenge to the limited hardware resources of these personal devices.

## Figures and Tables

**Table 1:** Characteristics of the smartphones employed in the testbed.

	Vendor/Model			
Characteristics	LG G4	Samsung Galaxy S6 (SG S6)	Samsung Galaxy A5 2017 (SG A5)	Xiaomi Mi A2
Launch date	June 2015	March 2015	January 2017	July 2018
Android version	6.0 (Marshmallow)	7.0 (Nougat)	8.0 (Oreo)	9.0 (Pie)
Processor	Qualcomm Snapdragon 808	Samsung Exynos 7420	Samsung Exynos 7870	Qualcomm Snapdragon 660
RAM (GB)	3	3	3	4
Bluetooth version	4.0	4.1	4.2	5.0
Battery capacity (mAh)	3000	2550	3000	3030

**Table 2:** Percentage of lost measurements for a sampling rate of 100 Hz and when just one sensing node is integrated in the BSN.

	Vendor/Model			
	LG G4	Samsung Galaxy S6 (SG S6)	Samsung Galaxy A5 (SG A5)	Xiaomi Mi A2
**Percentage of lost messages**	24.70%	0.86%	0.19%	0.03%

**Table 3:** Percentage of lost measurements for the LG G4 and Samsung Galaxy S6 using a sampling rate of 50 Hz as a function of the number of sensing points.

	Number of Sensing Nodes					
	**1**	**2**	**3**	**4**	**5**	**6**
LG G4	0.45%	0.46%	0.48%	0.72%	1%	0.74%
Samsung Galaxy S6	0.36%	0.52%	0.0002%	0.93%	0.87%	0%

**Table 4:** Number of received and lost messages per smartphone model. Tests were driven using a sensor module (with a sensing period of 20 ms) and running the YouTube app at the same time.

Vendor/Model	Number of Received Messages	Percentage of Lost Messages
LG G4	14879	0.00013%
Samsung Galaxy S6	14846	0%
Samsung Galaxy A5 (2017)	14768	0.00054%
Xiaomi Mi A2	14899	0%
